# Are House Prices Affected by PM_2.5_ Pollution? Evidence from Beijing, China

**DOI:** 10.3390/ijerph19148461

**Published:** 2022-07-11

**Authors:** Wenhao Xue, Xinyao Li, Zhe Yang, Jing Wei

**Affiliations:** 1 School of Economics, Qingdao University, Qingdao 266071, China; xuewh@mail.bnu.edu.cn (W.X.); yz69env@163.com (Z.Y.); 2 Business School, Beijing Normal University, Beijing 100875, China; 3 Department of Atmospheric and Oceanic Science, Earth System Science Interdisciplinary Center, University of Maryland, College Park, MD 20742, USA; weijing_rs@163.com

**Keywords:** PM_2.5_ pollution, house price, China, educational resources, ordinary least squares

## Abstract

With the progress of high-quality development in China, residents have begun to focus on the air quality of their residential areas in an effort to reduce the health threats of air pollution. Gradually, the risk associated with air pollution has become an important factor affecting housing prices. To quantitatively analyze the impact of air pollution on house prices, panel data, including data for fine particulate matter (PM_2.5_) concentrations, house prices and other auxiliary variables from 2009 to 2018, were collected from 16 districts in Beijing, China. Based on this dataset, ordinary least squares (OLS), moderating effect and threshold effect models were constructed for empirical investigation. Within the studied decade, PM_2.5_ pollution shows a significant decreasing trend of −3.79 μg m^−3^ yr^−1^ (*p* < 0.01). For house prices, the opposite trend was found. The empirical results indicate that PM_2.5_ pollution has a negative effect on house prices and that every 1% increase in PM_2.5_ causes an approximately 0.541% decrease in house prices. However, the inhibition of PM_2.5_ on housing prices is moderated by regional educational resources, especially in areas with high education levels. In addition, per capita disposable income can also cause heterogeneities in the impact of PM_2.5_ on house prices, whereby the threshold is approximately CNY 101,185. Notably, the endogeneity problems of this study are solved by the instrumental variable method, and the results are robust. This outcome suggests that the coordinated control of air pollution and balanced educational resources among regions are required for the future sustainable development of the real estate market.

## 1. Introduction

At present, urbanization and economic growth are accelerating in China, especially in metropolises and city clusters. However, China’s rapid modernization has been accompanied by serious air pollution, a problem closely related to human health [1,2,3]. Among all air pollutants, fine particulate matter (with an aerodynamic diameter of less than 2.5 μm, PM_2.5_) is particularly harmful. Long-term exposure to high PM_2.5_ loading significantly increases the risk of developing cancer as well as cardiovascular and respiratory diseases [4,5]. In addition, PM_2.5_ pollution poses a threat to ecological security [6,7,8]. At the same time, in addition to providing feedback regarding the quality of the atmospheric environment, such pollution can affect the living circumstances of urban residents [9,10]. Especially for areas experiencing high economic development, the economic losses caused by air pollution are substantial [11,12]. Meanwhile, with the increase in media publicity regarding the problem and rising income levels, low air pollution risk has become a goal for urban Chinese people. An increasing number of people tend to live in areas with low PM_2.5_ loading, resulting in counter urbanization, which has become a new challenge for environmental management and urban planning at the regional and national scales [13].

For urban residents, housing conditions are the foundation of high-quality development. Since the financial crisis of 2008, a long-term housing boom has appeared in China. According to the statistical data from the National Bureau of Statistics, the total sales of commercial housing in China were only approximately CNY 4399.5 billion in 2009. In contrast, they surpassed CNY 14,997.3 billion in 2018, accounting for ~16.3% of the gross domestic product (GDP). During this period, house prices increased substantially in China’s megacities, especially in Beijing, and real-averaged house prices increased by ~41.8% from 2012 to 2015 [14]. There are many factors that cause fluctuations in house prices in China [15,16,17,18,19]. Liu et al. used a demand-supply framework and annual data from 31 provinces across China from 2000 to 2018 to argue that five variables (i.e., land prices, real estate developer loans, per capita savings and the proportion of individuals with college or higher educational degrees) accounted for ~72% of the increase in house prices [20]. In addition, other phenomena, such as education level, entertainment facilities, wedding time, monetary factors and policy orientation, have been found to be potential influencing factors in China [21,22,23,24,25].

Since 2015, concomitant with the proposal by the United Nations of sustainable development goals (SDGs), the Chinese government has attached substantial importance to environmental protection [26]. Meanwhile, the awareness of environmental protection has gradually improved at the government, enterprise and individual scales. At present, environmental risk factors have gradually been incorporated into the considerations of house buyers. Subsequently, several researchers have examined the negative externality of the environmental quality of residential areas with respect to house prices [27,28,29,30]. Among all potential influence factors, air pollution, especially for PM_2.5_ pollution, plays an important role in house prices. Dai et al. found that higher PM_2.5_ risks were accompanied by lower house prices in Nanjing, China [9]. Similarly, the negative effects of PM_2.5_ concentration on housing prices were also found across cities in China, and the heterogeneity is also captured [31]. Sun and Yang, using the quantile regression and geographically weighted quantile regression, found the presence of asymmetric and spatial non-stationary effects among PM_2.5_ and housing prices in China [32]. Furthermore, the neighboring pollution also can lead to changes in local home prices [33]. Despite this wealth of literature, the mechanism of the effect of air pollution on the real estate market is less analyzed. More importantly, with the proposal of the ‘three-child policy’ in 2021 [34], the relationship between housing prices and the environment has become a matter of concern for every family, one that bears importance on the healthy growth of children in China.

The goal of this study was to investigate the impact of PM_2.5_ pollution on house prices. For this purpose, we collected panel data in all of Beijing’s administrative divisions from 2009 to 2018, including data on house prices, PM_2.5_ concentrations and auxiliary variables. Based on these datasets, the spatiotemporal variations in PM_2.5_ pollution and house prices were analyzed in detail. Then, an ordinary least squares (OLS) model was used as an econometric model to quantify the impact of PM_2.5_ concentrations on house prices. In addition, considering actual local/regional circumstances, the moderating effects of educational resources are explained. Finally, the per capita disposable incomes of local residents were used as a threshold effect to accurately identify the heterogeneities of the impact of PM_2.5_ concentrations on house prices. This study will provide a strong scientific basis and literature reference for regional urban planning, environmental protection and the benign development of the real estate market.

## 2. Study Area

**Figure 1:**
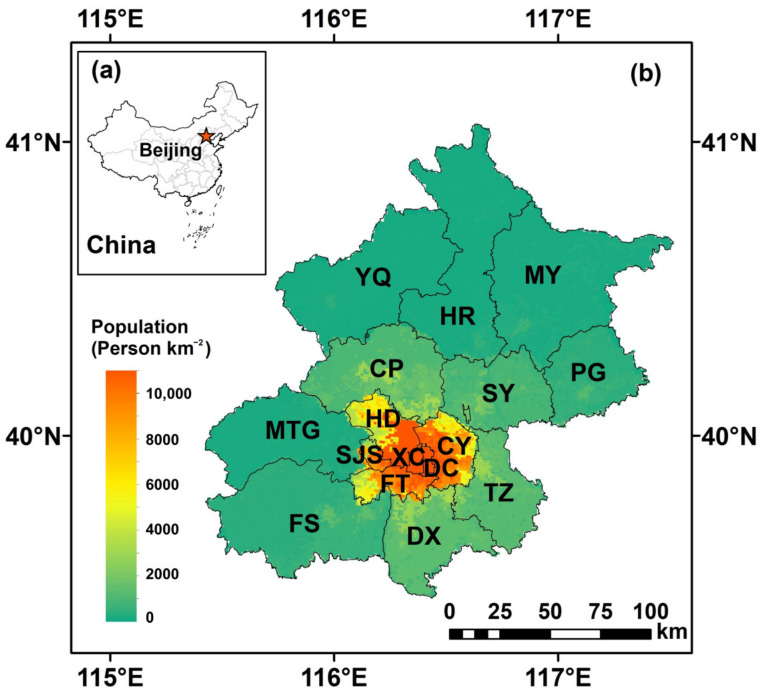
Beijing’s 16 administrative divisions. (**a**) is the geographical location of Beijing in China; (**b**) is the distribution of 16 districts in Beijing. The background colors indicate the distribution of population density (persons km^−2^); these data are available from the resource and environmental science and data center (https://www.resdc.cn/, accessed on 1 January 2022).

China’s capital, Beijing, is the country’s center of political, cultural, scientific and technological innovation. Figure 1 shows the geographical location of Beijing in northern China at longitudes and latitudes ranging from 115.7°–117.4° E and 39.4°–41.6° N, respectively. There are currently 16 administrative divisions in Beijing: Changping (CP), Chaoyang (CY), Daxing (DX), Dongcheng (DC), Fangshan (FS), Fengtai (FT), Haidian (HD), Huairou (HR), Mentougou (MTG), Miyun (MY), Pinggu (PG), Shijingshan (SJS), Shunyi (SY), Tongzhou (TZ), Xicheng (XC) and Yanqing (YQ). The CY, DC, FT, HD, SJS and XC districts are usually identified as the central urban areas. Figure 1 also shows the distribution of population density in Beijing. Generally, high population densities were captured in the central urban areas. The highest population density was captured in the XC district, with a value of 26,603 persons km^−2^, followed by the DC, HD, CY, SJS and FT districts, with mean population densities ranging from 7385 to 19,946 persons km^−2^. In contrast, the population density in the YQ district is the lowest (~173 persons km^−2^).

## 3. Dataset and Methodology

### 3.1. Dataset

#### 3.1.1. House Prices

The annual average house prices (unit: yuan) were collected in the 16 districts of Beijing from 2009 to 2018. These data were sourced from Anjuke, Inc., a real estate information service enterprise in China. The data are available at https://www.anjuke.com/fangjia/ (accessed on 1 January 2022). However, because of inflation effects, authentic house prices will be overvalued. Therefore, to calculate the authentic house prices of each district in Beijing, we used the provided house prices divided by a GDP deflator (i.e., the ratio of nominal GDP to real GDP).

#### 3.1.2. PM_2.5_ Concentrations

The ground-level PM_2.5_ concentrations were collected from the ChinaHighPM_2.5_ datasets (http://doi.org/10.5281/zenodo.3987359, accessed on 1 January 2022), with a high horizontal resolution of 1 km. In this dataset, the daily PM_2.5_ concentrations were generated using a linear mixed effect (LME) model combined with the Moderate Resolution Imaging Spectroradiometer (MODIS) Multi-Angle Implementation of Atmospheric Correction (MAIAC) aerosol optical depth products and meteorological factors, including boundary layer height (BLH), evaporation (ET), total precipitation (PRE), relative humidity (RH), surface pressure (SP), 2-m temperature (TEM), wind direction (WD) and wind speed (WS), which are potentially relevant variables in PM2.5 pollution. The build processes were explained in our previous study in detail [35]. Here, we extracted the PM_2.5_ concentrations of the corresponding pixels in each district of Beijing from 2009 to 2018. To avoid errors, we omitted pixels with values missing for more than ten days. Then, the monthly and annual concentrations were collected to analyze PM_2.5_ temporal trends and the impacts on house prices, respectively.

#### 3.1.3. Control Variables

According to previous research, greening facilities and socioeconomic factors can affect house prices. The descriptive statistics of all the control variables used in our study are listed in Table 1. In addition to annual PM_2.5_ concentrations, we selected seven other indices as control variables to reflect the greening facilities and socioeconomic status of all districts in Beijing during 2009–2018. These variables include GDP, the gross output value of residential services and other services (Service), per capita disposable income (Income), the gross output value of the construction industry (Industry), the normalized difference vegetation index (NDVI), the registered population (Population) and the number of private cars (Traffic). Similar to house prices, the sample capacities of all the control variables are 160 samples. In addition, the mean values and the standard deviation (Std) of all the control variables are provided. All other control variables were drawn from the regional statistical yearbook of Beijing with the temporal and spatial resolution of the district and annual level. In particular, in order to eliminate the influence of the heteroscedasticity of the model, we logarithmize all the continuous variables. Meanwhile, we made a collinearity diagnosis. The Variance Inflation Factor (VIF) values are all less than 10, which indicates that there is no collinearity problem [36].

#### 3.1.4. Other Variables

Due to the uneven distribution of urban educational resources, a derivative, i.e., school district housing, occurs in the real estate market across Beijing, which could also impact house prices on a local scale. Therefore, to investigate the moderating effect of educational resources on the relationship between PM_2.5_ and house prices, the gross regional product of education from 2009 to 2018 in all districts was selected as the index to reflect the education level. These data were collected from the regional statistical yearbook of Beijing. Furthermore, to avoid the endogeneity problems caused by missing variables, the temperature was selected as the instrumental variable. Temperature can affect the generation and diffusion of PM_2.5_. In addition, it has no direct relationship with house prices. Here, the annual averaged temperature during 2009–2018 was adopted from the fifth-generation European Center for Medium-Range Weather Forecasts (ECMWF) atmospheric reanalysis dataset of the global climate (ERA5), with a horizontal resolution of 0.25° × 0.25° [37].

### 3.2. Methodology

#### 3.2.1. Benchmark Regression Model

To authenticate the test of the impact of PM_2.5_ concentrations on house prices, we selected the OLS model as the benchmark regression model. This model is a mature model in econometrics, environmental economics and other economic-related research, and it was proven to be one of the econometric models that can identify the causal relationship of variables [38,39,40,41,42,43,44,45,46]. Meanwhile, considering the possible endogeneity problem of the model and in order to compare the results of similar models, we selected temperature as an instrumental variable and used the two-stage least squares (2SLS) method to test and compare the results, which can also prove the robustness of our model [47]. The OLS model was set as follows.

(1) $$\begin{array}{c} HP_{it}=\alpha_{0}+\alpha_{1}{\mathrm{PM}}_{2.5_{it}}+\alpha_{2}{\mathrm{GDP}}_{it}+\alpha_{3}{\mathrm{Service}}_{it}+\alpha_{4}{\mathrm{Income}}_{it}+\alpha_{5}{\mathrm{Industry}}_{it}\\ +\alpha_{6}{\mathrm{NDVI}}_{it}+\alpha_{7}{\mathrm{Population}}_{it}+\alpha_{8}{\mathrm{Traffic}}_{it}+\varepsilon 1_{it} \end{array}$$

where i indicates the district, and t indicates the year. *HP* represents house prices. PM_2.5_ indicates the regional average PM_2.5_ concentration. The coefficient $\alpha_{1}$ captures the effect of PM_2.5_ on house prices. $\alpha_{0}$ is the constant term, and $\varepsilon 1_{it}$ is the error term. $\alpha_{2}$, $\alpha_{3}$, …, $\alpha_{8}$ represent the effects of other control variables on house prices, including GDP, Service, Income, Industry, NDVI, Population and Traffic.

#### 3.2.2. Moderating Effect Models

We further explain the regulating role of education in the relationship between PM_2.5_ concentration and house prices. A moderating variable, i.e., education, was selected and added to the basic econometric model. In addition, the interaction term of education and PM_2.5_ is calculated as a new explanatory variable in Equation (2).

(2) $$\begin{array}{l} HP_{it}=\beta_{0}+\beta_{1}{\mathrm{PM}}_{2.5_{it}}+\beta_{2}{\mathrm{GDP}}_{it}+\beta_{3}{\mathrm{Service}}_{it}+\beta_{4}{\mathrm{Income}}_{it}+\beta_{5}{\mathrm{Industry}}_{it}\\ \;\;\;\;\;\;\;\;\;\;\;\;\;\;\;\;\;\;\;\;\;\;\;\;\;\;\;\;\;\;+\beta_{6}{\mathrm{NDVI}}_{it}+\beta_{7}{\mathrm{Population}}_{it}+\beta_{8}{\mathrm{Traffic}}_{it}+\beta_{9}{\mathrm{Education}}_{it}\\ \;\;\;\;\;\;\;\;\;\;\;\;\;\;\;\;\;\;\;\;\;\;\;\;\;\;\;\;\;\;+\beta_{10}{\mathrm{Education}}_{it}\times {\mathrm{PM}}_{2.5_{it}}+\varepsilon 2_{it} \end{array}$$

where ${\mathrm{Education}}_{it}$ refers to the education level in district i in year t. $\beta_{0}$ is the constant term. $\beta_{1}$, $\beta_{2}$, …, $\beta_{9}$ represent the effects of the control variables on house prices. In addition to the control variables in Equation (1), education was also considered here. ${\mathrm{Education}}_{it}\times {\mathrm{PM}}_{2.5_{it}}$ is the interaction term of education and PM_2.5_. $\beta_{10}$ indicates the coefficient of the regulatory effect of education. $\varepsilon 2_{it}$ is the error term.

(3) $$\frac{\partial {\left(HP\right)}_{it}}{\partial {\left({\mathrm{PM}}_{2.5}\right)}_{it}}\;=\;\beta_{1}+\beta_{10}{\mathrm{Education}}_{it}$$

Then, by deriving PM_2.5_ as Equation (3), the boundary effects of education on the relationship between PM_2.5_ and house prices are quantified, whereby $\beta_{1}$ represents the direct effect of PM_2.5_ on house prices, and $\beta_{10}$ indicates the interactions.

#### 3.2.3. Threshold Effect

**Figure 2:**
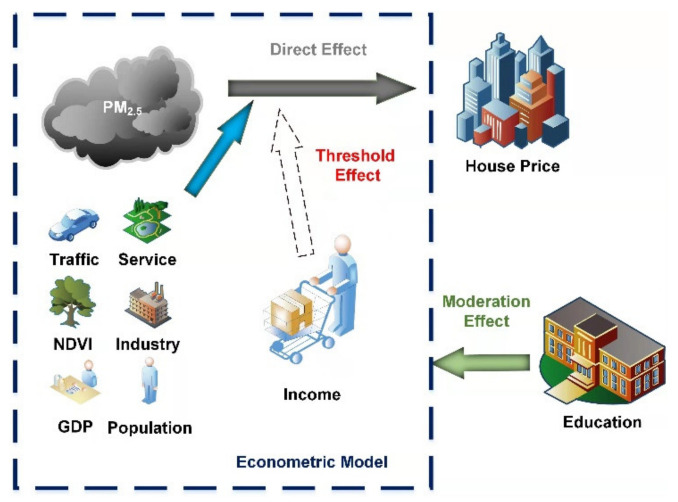
The analytical framework for the impact of PM_2.5_ on house prices.

However, the relationship between PM_2.5_ and house prices may be nonlinear, and the regional per capita disposable income level may be an important determinant. Therefore, we use a threshold effect model and adopt per capita disposable income as a threshold variable to portray this relationship. The threshold effect model was established as follows:

(4) $$\begin{array}{l} HP_{it}\;=\;\gamma_{0}+\gamma_{1}{\mathrm{PM}}_{2.5}{\left(thr<\theta \right)}_{it}+\gamma_{2}{\mathrm{PM}}_{2.5}{\left(thr⩾\theta \right)}_{it}+\gamma_{3}{\mathrm{GDP}}_{it}+\gamma_{4}{\mathrm{Service}}_{it}\\ \;\;\;\;\;\;\;\;\;\;\;\;\;\;\;\;\;\;\;\;\;\;\;\;\;\;\;\;\;\;+\gamma_{5}{\mathrm{Industry}}_{it}+\gamma_{6}{\mathrm{NDVI}}_{it}+\gamma_{7}{\mathrm{Population}}_{it}+\gamma_{8}{\mathrm{Traffic}}_{it}\\ \;\;\;\;\;\;\;\;\;\;\;\;\;\;\;\;\;\;\;\;\;\;\;\;\;\;\;\;\;\;+\varepsilon 3_{it} \end{array}$$

where *thr* represents the threshold variable, and θ is the estimated threshold value. $\gamma_{0}$ indicates the constant term. $\gamma_{1}$ and $\gamma_{2}$ represent the influence coefficient of PM_2.5_ on the dependent variable house prices under the level of per capita disposable income <θ and ⩾θ in district i in year t, respectively. $\gamma_{3}$, $\gamma_{4}$ … $\gamma_{8}$ represent the effects of the control variables on house prices. The control variables include GDP, Service, Industry, NDVI, Population and Traffic. $\varepsilon 3_{it}$ is the error term. The overall framework for analyzing the impact of PM_2.5_ on house prices is shown in Figure 2.

#### 3.2.4. Temporal Trend and Correlation Analysis

To examine PM_2.5_ pollution characteristics in the BTH region, we used the monthly deseasonalized temporal trend analysis method, first calculating the monthly anomalous PM_2.5_ concentrations at a horizontal resolution of 1 km. Then, the linear PM_2.5_ trend was calculated in each pixel based on the OLS fitting method [48]. To evaluate the accuracy of the temporal trend analysis, a paired-samples T-test was used. In addition, to confirm the relationship between the control variables and house prices, the Pearson correlation coefficient (r) was calculated before the construction of the econometric model. The T-test was selected as the significance level test in this study.

## 4. Results and Discussion

### 4.1. Spatiotemporal Characteristics of PM_2.5_ and House Prices

**Figure 3:**
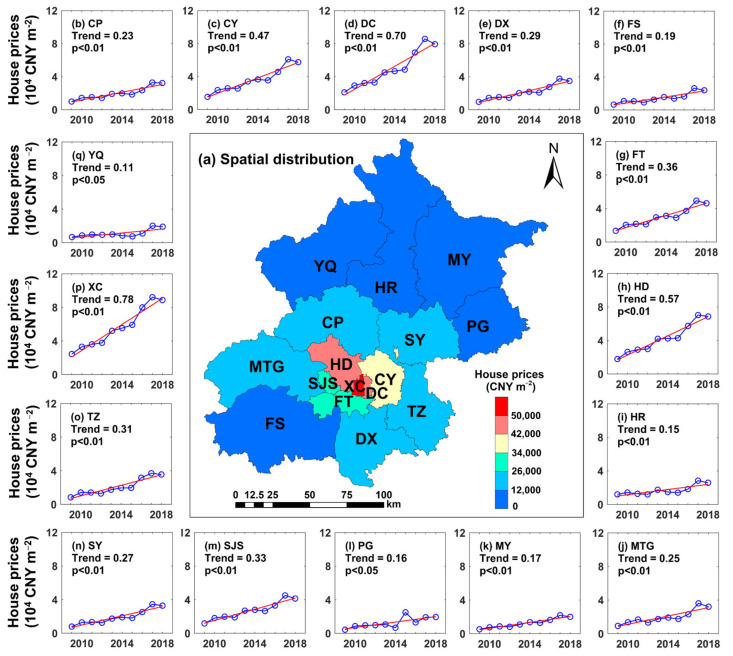
Spatiotemporal characteristics of house prices across Beijing from 2009 to 2018. (**a**) is the spatial distribution; (**b**–**q**) represent the temporal changes in the 16 districts.

Figure 3a shows the spatial distribution of annual average house prices across Beijing from 2009 to 2018. Generally, the mean house price in Beijing is approximately CNY 25,654.34 m^−2^. However, significant spatial heterogeneities in house prices occur in the city. The high-level house price areas are mainly concentrated in the central urban districts, with a mean value of CNY 40,088.39 m^−2^. Among all the districts, high values were captured in the XC, DC and HD districts, with house prices of CNY 55,899.58 m^−2^, CNY 49,026.04 m^−2^ and CNY 42,665.04 m^−2^, respectively. In contrast, low-level house price areas are found in the YQ, MY and PG districts, with values of CNY 11,077.95 m^−2^, CNY 12,603.18 m^−2^ and CNY 12,681.34 m^−2^, respectively. Figure 3 also shows the temporal variation characteristics of house prices in Beijing during 2009–2018. With the development of the local economic level and changes in the supply-demand relationship, significant increasing trends are found in every district. Generally, the average house prices in Beijing increased by approximately 2.58 times in one decade, and the increasing trend was CNY 3350.0 m^−2^ per year^−1^. Similarly, the increasing rates of each district are quite different. The largest increase was captured in the XC district, with a trend of approximately CNY 7754.7 m^−2^ per year^−1^ (an increase of 2.63 times). The YQ district exhibited the lowest enhancement—the increasing trend was only CNY 1143.9 m^−2^ per year^−1^. Nevertheless, in the YQ district, the house prices in 2018 were still 2.84 times those in 2009. Throughout the past decade, there have been two periods of accelerated increase: 2009–2010 and 2012–2013, in which the mean house price growth in Beijing was 47.0% and 35.6%, respectively. Notably, reflecting the market and policy regulation mechanism, two periods of decrease are captured, with decreases of 3.0% and 5.7% during 2011–2012 and 2017–2018, respectively.

**Figure 4:**
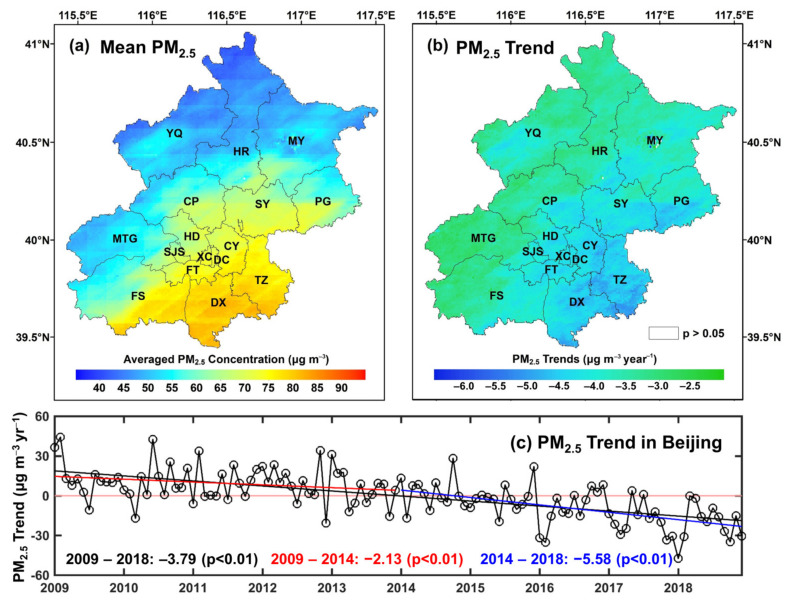
Annual averaged concentrations and trends in PM_2.5_ distributions across Beijing from 2009 to 2018. (**a**) is the spatial distribution of annual averaged PM_2.5_; (**b**) is the spatial distribution of the temporal trends; (**c**) is the overall trend of PM_2.5_ concentration in Beijing.

Figure 4 shows the mean PM_2.5_ spatial distributions during the study period in Beijing. Generally, the annual average PM_2.5_ concentration is 67.33 ± 15.95 μg m^−3^, and in nearly all of the districts, it was higher than 35 μg m^−3^ (the second level of the ambient air quality standards in China). The highest average concentration can reach approximately 83.18 μg m^−3^. However, the concentration changed obviously on a spatial scale and was extremely high in the southern regions, especially in the DX district (82.38 ± 14.81 μg m^−3^), followed by the TZ district (79.80 ± 15.12 μg m^−3^). Conversely, low PM_2.5_ loading was captured in the northern regions, especially in the YQ, HR and MY districts, with annual averaged PM_2.5_ concentrations of 49.95 ± 11.64 μg m^−3^, 51.05 ± 12.12 μg m^−3^ and 54.16 ± 12.97 μg m^−3^, respectively. Significantly, the spatial pattern of PM_2.5_ concentrations in Beijing is consistent with the topography and distribution of the secondary industry [35]. Figure 4 also presents the temporal trends of PM_2.5_ concentrations. Overall, the PM_2.5_ concentrations decreased significantly across Beijing during this period, with a trend of −3.79 μg m^−3^ yr^−1^ (*p* < 0.01). An accelerated decreasing trend was captured after 2014 (−5.58 μg m^−3^ yr^−1^, *p* < 0.01), which reflects the implementation of the Air Pollution Prevention and Control Action Plan in the Beijing-Tianjin-Hebei region [49]. Regarding the spatial distributions of temporal trends, most Beijing districts show significant decreasing PM_2.5_ pollution (*p* < 0.05), especially in the southeastern region, i.e., the DX (−4.46 μg m^−3^ yr^−1^, *p* < 0.01), TZ (−4.64 μg m^−3^ yr^−1^, *p* < 0.01) and CY (−4.41 μg m^−3^ yr^−1^, *p* < 0.01) districts, respectively. In contrast, slight weakening trends were observed in several western areas in Beijing (e.g., the MTG and CP districts).

### 4.2. Spatial Correlation

In addition, we also calculated the spatial relationship between PM_2.5_ and HP. Firstly, we vectorized the geographical map of the Beijing region to obtain latitude and longitude information. Then, we constructed the regional inverse distance weight matrix as Equation (5). It assumes that the strength of the spatial effect depends on the distance, and the closer the spatial effect between spatial units is, the stronger the spatial effect. $W_{ij}$ indicates the inverse distance weight matrix, and $d_{ij}$ is the distance for each district.

(5) $$W_{ij}=\left\{\begin{array}{c} \frac{1}{d_{ij}{}^{2}}\\ 0 \end{array}\right.$$

Then, the spatial autocorrelation analysis was used in this study to verify the spatial correlation between PM_2.5_ and house prices in Beijing. The PM_2.5_ concentration of different districts may have some spatial correlation for two main reasons. First, PM_2.5_ in one region will diffuse to other districts through atmospheric transport. Second, PM_2.5_ may receive shocks from other non-environmental factors such as economic and political factors and thus exhibit spatial correlation. Therefore, the global Moran’s I is used to study the overall spatial correlation as follows:

(6) $$I=\frac{\sum_{i=1}^{n}\sum_{j=1}^{n}W_{ij}\left(X_{i}-\bar{X}\right)\left(X_{j}-\bar{X}\right)}{S^{2}\sum_{i=1}^{n}\sum_{j=1}^{n}W_{ij}}$$

where *n* is the number of districts, and $X_{i}$ and $X_{j}$ are the PM_2.5_ of district *i* and district *j*, respectively. $\bar{X}$ is the average distance among all districts. $W_{ij}$ is the spatial weight matrix, and $s^{2}$ is the variance value of PM_2.5_. At a certain level of significance, the larger the absolute value of Moran’s I is, the higher the spatial correlation is. The significance of Moran’s I was tested as follows:

(7) $$Z_{I}=\frac{I-E\left[I\right]}{\sqrt{V\left[I\right]}}$$

where $E\left[I\right]$ is the expectation of Moran’s I and $V\left[I\right]$ denotes the standard deviation of the variable. Meanwhile, the spatial autocorrelation of house prices was also explored (Table 2). The Moran’s I values are all positive (*p* < 0.01), which indicates that the PM_2.5_ distribution in different areas has a high spatial correlation. The spatial autocorrelation of house prices also shows the same characteristics. Therefore, it can be seen that PM_2.5_ and house prices have a spatial correlation.

### 4.3. Correlation Analysis

**Figure 5:**
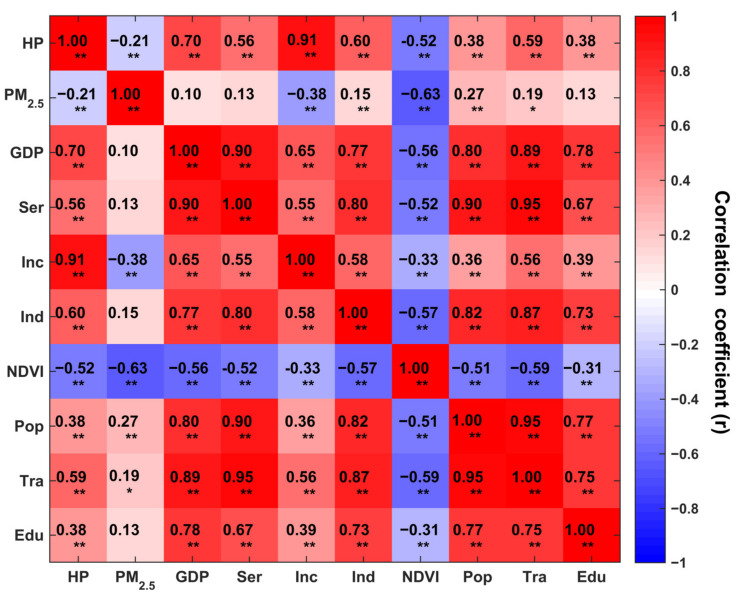
Correlations among house prices, control variables and moderating variables. * and ** indicate significance levels of *p* less than 0.05 and 0.01, respectively.

Figure 5 shows the r values between house prices and the other variables used. Generally, the r values between the control variables and house prices are all significant (*p* < 0.01). Here, PM_2.5_ and NDVI show a negative correlation with house prices, with coefficients of −0.21 (*p* < 0.01) and −0.52 (*p* < 0.01), respectively, which proves that there are associations between house prices and air pollution. Notably, the correlation coefficients among the control variables are significant, but the values are heterogeneous, indicating that the other variables exist independently from one another to varying degrees and can be used for modeling here.

### 4.4. Impact of PM_2.5_ on House Prices

Table 3 lists the regression results of the OLS estimation. The results indicate that the PM_2.5_ concentration could reduce house prices when other control variables (e.g., GDP, Service, Income) are controlled, and the significance *p* is at the 1% level. Specifically, according to the estimation coefficient of our econometric model, when the annual mean PM_2.5_ concentration increases by 1%, the average house prices decrease by 0.541% across Beijing. As the economy continues to develop rapidly, environmental pollution is becoming increasingly serious, resulting in an increasing marginal cost caused by environmental pollution. Currently, although rapid economic development at the expense of the environment can improve the material consumption level of residents, it can also offset the satisfaction of local residents brought by the increase in economic income. Due to the negative externality of the environment, house prices will decline. Currently, residents favor residential districts with better air quality to avoid the health risk of long-term exposure to air pollution. This phenomenon can be explained by the strong susceptibility of residents to the air pollution level of their residential area. During our study period, although the PM_2.5_ concentration showed a significant decreasing trend, it remained at a high level. This phenomenon enhances the demand for housing with higher environmental quality by local residents, which will eventually promote the rise in house prices.

The regression results for the other control variables also have practical significance. Here, significantly positive coefficients are captured for GDP, Income and Industry. Every 1% increase in GDP will lead to an increase in house prices of 0.128% (*p* < 0.01). This result illustrates the direct impact of regional economic strength on house prices, which is consistent with previous studies [50]. Regarding Income, the estimated coefficient is approximately 1.020 (*p* < 0.01), indicating that the improvement of per capita disposable income will significantly increase the pursuit of living quality for local residents. Notably, a 1% increase in the gross output value of the construction industry will also raise house prices by approximately 0.101% (*p* < 0.05). As a megacity in northern China, Beijing, with its high-quality social and public resources and good employment opportunities, exerts a strong attraction to the floating population. However, increasing the floating population could also cause a housing shortage. Moreover, the space available for housing in the central urban area is limited, resulting in numerous real estate developments in surrounding areas. Meanwhile, the construction cost of housing is also enhanced. These two factors will eventually lead to higher home prices. In contrast, significant negative impacts were captured for NDVI and Population with respect to house prices, with coefficients of −0.879 (*p* < 0.01) and −0.212 (*p* < 0.01), respectively. The areas with high NDVIs are mainly concentrated in Beijing’s suburban regions, while the house prices in those areas are generally low, resulting in negative effects. The registered population is closely related to local house price control policies, e.g., preferential policies for the first house, price limits and purchase restrictions, which can encourage highly skilled individuals to settle in the locality.

We also found that the estimated coefficients of Service and Traffic are not significant. Nevertheless, they still possess practical significance. Following the implementation of a policy to strengthen livelihoods, the investment in public facilities in all districts has increased, and local residents enjoy good infrastructure and services across Beijing. Therefore, Service is no longer a factor that must be considered when purchasing a house. Regarding Traffic, on the one hand, because of the increase in per capita GDP, private car ownership generally increased across all districts during the study period, resulting in the improvement of the convenience of local resident travel. On the other hand, with the construction of basic transportation services, public transportation is available in all of Beijing’s districts. These two items indicated that the traffic situation is no longer the main factor affecting house prices in the city.

### 4.5. Moderating Effect of Education

**Figure 6:**
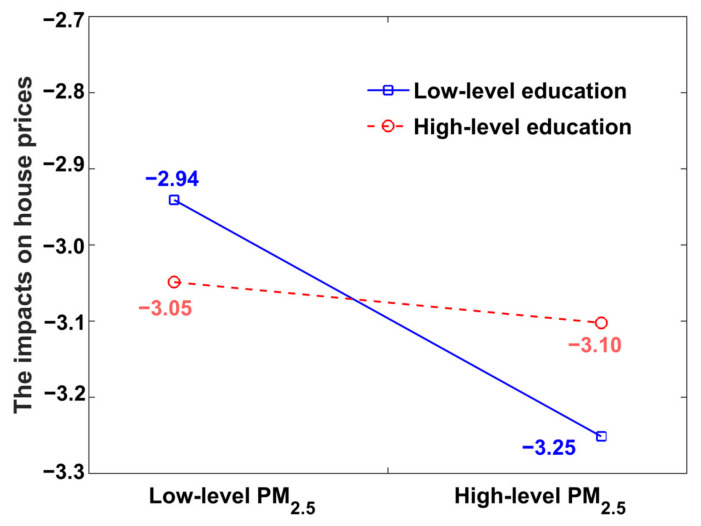
Moderating effect of education on the impact of PM_2.5_ on house prices.

Table 3 also shows the results of the moderating effect of education on the relationship between PM_2.5_ and house prices. Here, the estimation coefficient of PM_2.5_ concentrations on house prices is negative, with a significance level of *p* < 0.05. In contrast, the estimation coefficient interactive item of Education and PM_2.5_ was significantly positive (*p* < 0.01), indicating that educational resources may positively adjust the relationship between house prices and PM_2.5_ concentrations. Specifically, local residents will be more inclined to allocate their purchasing power to housing that includes access to superior educational resources, resulting in the enhancement of house prices near preferred school districts. Figure 6 intuitively shows the moderating mechanism of education level on the relationship between PM_2.5_ pollution and house prices. Generally, PM_2.5_ concentrations can cause decreases in house prices, and the reduction will increase with the aggravation of the PM_2.5_ pollution level. However, with the moderating influence of educational resources, this negative effect will be relieved effectively. In addition, the mitigation will be enhanced with an increase in the level of educational resources. During the study period, the policy for the next generation of the compulsory education stage (before high school) in Beijing mainly consisted of students attending schools nearby their homes. This policy not only strengthens the equity of educational resource allocation but also makes the location of residential areas the decisive factor in the allocation of basic educational resources. Currently, education level is a primary development factor for the younger generation. Therefore, houses with access to high-level educational resources have become popular among Beijing homebuyers. Such houses, which can be termed “school district houses”, eventually result in increased house prices for the residents of the preferred districts.

### 4.6. Threshold Regression Result

Significant heterogeneities may occur in the relationship between PM_2.5_ pollution and house prices. Therefore, per capita disposable income was selected as a threshold variable to explain those heterogeneities. Initially, to determine the threshold effect, we first estimated the threshold condition according to Formula 4 under single, double and triple thresholds. Then, the joint hypotheses test (F statistic) was used to determine whether the model parameters were suitable for estimation. Additionally, the significance level *p* was calculated according to the bootstrap method [51]. Overall, only a single threshold effect was statistically significant and reached a significant level. Table 4 shows the test results of the single threshold regression. Here, the F statistic and relevant critical values in this table are the results of repeated sampling (1000 times) with the bootstrap method. The F statistic was significant in the single threshold model, with the *p* value at the 5% significance level (*p* = 0.017). Furthermore, the threshold value of per capita disposable income *θ* was calculated to be approximately CNY 101,185.

Table 3 shows the results of the threshold regression. Obvious differences in the impact of PM_2.5_ pollution on house prices were captured under high- and low-level per capita disposable income. Generally, the estimation coefficients are both significantly negative, indicating that PM_2.5_ pollution will reduce house prices. However, significant differences occurred in the quantitative estimation. When per capita disposable income is less than *θ* (Income < CNY 101,185), for every 1% increase in PM_2.5_ concentration, house prices will decrease by 0.425%. Then, with the growth of per capita disposable income (Income ≥ CNY 101,185), the impact of PM_2.5_ pollution on house prices will intensify. Under such circumstances, 1% PM_2.5_ pollution aggravation can cause house prices to decrease by approximately 0.461%. Higher per capita disposable income often means that residents will spend more on improving their quality of life. High PM_2.5_ pollution loading can seriously threaten human health, which can increase the health risks for surrounding residents. At the same time, the awareness of environmental risks among local residents is also gradually growing. Therefore, they may spend more money to live in an environment with lower air pollution loading, resulting in changes in the relationship between PM_2.5_ concentrations and house prices in Beijing.

### 4.7. Robustness Test

#### 4.7.1. Endogeneity Problems

Although we attempted to reduce the endogeneity problems by using control variables, the bias of omitted variables could not be completely avoided, which may lead to deviations in the estimation results. Therefore, the instrumental variable method was used to overcome the influence of endogeneity in our econometric model. The annual mean temperature was selected as the instrumental variable, and a 2SLS model was constructed. The first and second columns of Table 5 show the results of the 2SLS model. Generally, the instrumental variable is valid, and its estimation coefficient of the regression is significantly negative in the first stage (*p* < 0.05). A temperature increase can lead to an increase in the mixing layer height, which creates good air-diffusion conditions. With an intensification of atmospheric flow, air pollution is diluted, eventually resulting in a decrease in PM_2.5_ concentration. In addition, the F value is greater than 10 and at the significance level of the 1% confidence interval, indicating that no weak instrumental variable problem occurred in the model. Among the results of the two-stage regression, the estimation coefficient of PM_2.5_ is also significantly negative (*p* < 0.05). This outcome means that PM_2.5_ pollution can still decrease housing prices even when fully considering the influence of the housing price background and control variables. Specifically, for every 1% increase in PM_2.5_ concentration, house prices will decrease by approximately 0.897% across Beijing. Compared with the results of the basic econometric model, the estimation coefficient of the 2SLS model is 1.66 times that of the OLS model, indicating that the OLS model may slightly underestimate the impacts of PM_2.5_ concentrations on house prices. In addition, the positive and negative conditions for the estimated coefficients of the other control variables in the 2SLS model have not changed, which can also prove the robustness of the results obtained in our study.

#### 4.7.2. Winsorized Robust Measures

Winsorization was also used to further determine the robustness of our results. Because of the limited sample size, we winsorized 3% of the samples. The third column of Table 5 shows the results of the winsorized robustness test. Overall, our econometric model is robust, and the positive and negative characteristics of the estimated coefficient of all controls are consistent with the results of the basic regression. Additionally, the level of significance did not change. Only the quantification of the estimated coefficients changed, extremely subtly in our case. In summary, combining the results of the IV method and the winsorized robustness test, our econometric model is robust. The impact of PM_2.5_ pollution on house prices in Beijing was real during the study period.

## 5. Conclusions

At present, health risks, especially PM_2.5_ pollution risks, in residential areas have gradually become the focus of local residents in China. In this study, the panel data for 16 districts in Beijing from 2009 to 2018 were used to investigate the impact of PM_2.5_ pollution on house prices from theoretical and empirical perspectives. Through an econometric model analysis, we found that PM_2.5_ pollution can curb the increase in house prices in Beijing. For every 1% increase in annual mean PM_2.5_ concentration, house prices decrease by 0.541%. However, this impact can be suppressed by the moderating effects of the level of education. Moreover, as educational resources increase, this moderating effect will gradually increase. Meanwhile, the effects of PM_2.5_ concentration on house prices are also nonlinear and influenced by disposable income per capita. When per capita disposable income is less than CNY 101,185, house prices will decrease by 0.425% for every 1% increase in PM_2.5_; otherwise, house prices will decrease by 0.460% for every 1% increase in PM_2.5_. Significantly, endogeneity problems are solved here by the instrumental variable method, and the conclusions of the paper are demonstrated to be robust.

## 6. Policy Suggestions

In this research, we provide a new perspective for understanding the economic consequences of air pollution. Our findings have important policy implications for promoting a win-win relationship between air pollution control and a stable real estate market across China. Based on our results, we offer several policy suggestions. First, the government should further optimize the environmental governance system and promote interregional collaborative governance. PM_2.5_ pollutants usually exert spillover effects. Therefore, it is necessary to establish an interregional joint prevention and control system to decrease air pollution. Second, local governments should establish a house price control system to strictly control the inefficient expansion of urbanization. Additionally, housing planning should be strengthened to promote the sustainable development of a livable environment. Through the rationalized control of house prices and the optimization of the spatial layout, the impact of air pollution on house prices can be reduced, and the win-win goal of green development and house price regulation can be achieved. Finally, the local government should balance educational resources among all districts of the city. The matching degree of the supply and demand of such resources should be improved, which can effectively reduce the spatial difference of house prices. These suggestions will contribute to achieving the high-quality development goals of balancing the housing price market, reducing resident health risks, reasonably improving the education level of the next generation and ameliorating urban planning.

## Figures and Tables

**Table 1:** The descriptive statistic of all control variables (*n* = 160).

Abbreviation	Control Variable	Unit	Mean	Std
PM_2.5_	Annual averaged concentration of PM_2.5_	μg m^−3^	67.33	15.95
GDP	Gross domestic product	Billion yuan	98.07	118.00
Service	Gross output value of residential services and other services	Million yuan	71,202.03	78,697.96
Income	Per capita disposable income	Yuan	32,551.19	9659.59
Industry	Gross output value of the construction industry	Million yuan	379.56	368.15
NDVI	Normalized Difference Vegetation Index	-	0.39	0.09
Population	Registered population	Thousand person	920.60	572.60
Traffic	Number of private cars	Set	254,223.80	226,015.90

**Table 2:** Results of the Moran test.

**Year**	**2009**	**2010**	**2011**	**2012**	**2013**
PM_2.5_	0.108 ***	0.081 ***	0.102 ***	0.105 ***	0.130 ***
HP	0.175 ***	0.206 ***	0.216***	0.208 ***	0.205 ***
**Year**	**2014**	**2015**	**2016**	**2017**	**2018**
PM_2.5_	0.101 ***	0.068 ***	0.108 ***	0.137 ***	0.138 ***
HP	0.211 ***	0.187 ***	0.203 ***	0.214 ***	0.211 ***

*** indicate *p* < 0.01.

**Table 3:** The regression results of the OLS estimation, moderating effect and threshold effect.

	(1)	(2)	(3)
	OLS Model	Moderating Effect	Threshold Regression
Variables	House Prices	House Prices	House Prices
PM_2.5_	−0.541 ***	−0.349 **	
	(−3.20)	(−2.14)	
GDP	0.128 ***	0.157 ***	1.140 ***
	(2.66)	(3.04)	(4.73)
Service	0.093	0.060	0.235 **
	(1.60)	(1.02)	(1.99)
Income	1.020 ***	1.085 ***	
	(8.10)	(8.63)	
Industry	0.101 **	0.110 ***	0.180 **
	(2.53)	(2.70)	(2.51)
NDVI	−0.879 ***	−0.784 ***	−1.041 ***
	(−6.51)	(−5.61)	(−2.62)
Population	−0.212 ***	−0.283 ***	−0.090
	(−4.14)	(−4.97)	(−1.42)
Traffic	−0.036	−0.009	0.228
	(−0.62)	(−0.13)	(1.46)
Education		0.005	
		(0.12)	
Education × PM_2.5_		0.243 ***	
		(3.00)	
PM2.5 (Income < *θ*)			−0.425 *
			(−1.93)
PM2.5 (Income ≥ *θ*)			−0.461 **
			(−2.08)
Constant	−1.423	−3.086 *	−12.622 ***
	(−0.83)	(−1.83)	(−3.11)
Observations	160	160	160
R-squared	0.901	0.906	0.897

***, ** and * indicate *p* < 0.01, *p* < 0.05 and *p* < 0.1.

**Table 4:** The test results of the threshold regression.

	F	*p*	RSS	MSE	Ctrit10	Ctrit5	Ctrit1
Single (*θ* = 101,185)	14.430	0.017	2.673	0.018	9.368	11.123	14.909

**Table 5:** The results of the robust test.

	(1)	(2)	(3)
	Stage1	Stage2	Robust
Variables	House Prices	House Prices	House Prices
PM2.5		−0.897 **	−0.513 ***
		(−2.20)	(−3.06)
GDP	0.183 ***	0.093 *	0.133 ***
	(4.06)	(1.71)	(2.82)
Service	0.027	0.119 *	0.085
	(0.45)	(1.89)	(1.51)
Income	1.314 ***	0.828 ***	1.040 ***
	(13.13)	(3.38)	(8.31)
Industry	0.091 *	0.131 **	0.099 **
	(1.95)	(2.34)	(2.49)
NDVI	−0.674 ***	−1.079 ***	−0.866 ***
	(−6.46)	(−4.38)	(−6.21)
Population	−0.331 ***	−0.152 **	−0.200 ***
	(−6.32)	(−2.10)	(−3.84)
Traffic	0.053	−0.066	−0.047
	(0.86)	(−0.92)	(−0.82)
Temperature	−14.255 **		
	(−2.10)		
Constant	73.330 *	2.058	−1.632
	(1.92)	(0.50)	(−0.95)
Observations	160	160	160
Cragg-Donald Wald F statistics	43.320	-	-
R-squared	0.898	0.897	0.904

***, ** and * indicate *p* < 0.01, *p* < 0.05 and *p* < 0.1.
